# Curcumin‐induced exosomal FTO from bone marrow stem cells alleviates sepsis‐associated acute kidney injury by modulating the m6A methylation of OXSR1

**DOI:** 10.1002/kjm2.12923

**Published:** 2024-12-30

**Authors:** Ting Yang, Hui Yu, Zheng Xie

**Affiliations:** ^1^ Department of Emergency Medicine Affiliated Hospital of Jiangnan University Wuxi China

**Keywords:** bone marrow stem cells, exosomes, FTO, OXSR1, sepsis‐associated AKI

## Abstract

Curcumin and bone marrow stem cells (BMSCs)‐derived exosomes are considered to be useful for the treatment of many human diseases, including sepsis‐associated acute kidney injury (SA‐AKI). However, the role and underlying molecular mechanism of curcumin‐loaded BMSCs‐derived exosomes in the progression of SA‐AKI remain unclear. Exosomes (BMSCs‐EXO^Curcumin^ or BMSCs‐EXO^Control^) were isolated from curcumin or DMSO‐treated BMSCs, and then co‐cultured with LPS‐induced HK2 cells. Cell proliferation and apoptosis were determined by cell counting kit 8 (CCK8) assay, 5‐ethynyl‐2‐deoxyuridine (EdU) assay, and flow cytometry. Enzyme‐linked immunosorbent assay (ELISA) was used for examining inflammatory factors. The levels of SOD, MDA, and ROS were tested to assess oxidative stress. The levels of fat mass and obesity‐associated protein (FTO) and oxidative stress responsive 1 (OXSR1) were detected by quantitative real‐time PCR and western blot. Methylated RNA immunoprecipitation (MeRIP) assay and RNA immunoprecipitation (RIP) assay were used for measuring the interaction between FTO and OXSR1. BMSCs‐EXO^Curcumin^ treatment could inhibit LPS‐induced HK2 cell apoptosis, inflammation, and oxidative stress. FTO was downregulated in SA‐AKI patients and LPS‐induced HK2 cells, while was upregulated in BMSCs‐EXO^Curcumin^. Exosomal FTO from curcumin‐induced BMSCs suppressed apoptosis, inflammation, and oxidative stress in LPS‐induced HK2 cells. FTO decreased OXSR1 expression through m6A modification, and the inhibitory effect of FTO on LPS‐induced HK2 cell injury could be eliminated by OXSR1 overexpression. In animal experiments, BMSCs‐EXO^Curcumin^ alleviated kidney injury in SA‐AKI mice models by regulating FTO/OXSR1 axis. In conclusion, exosomal FTO from curcumin‐induced BMSCs reduced OXSR1 expression to alleviate LPS‐induced HK2 cell injury and improve kidney function in CLP‐induced mice models, providing a new target for SA‐AKI.

## 1 INTRODUCTION

Sepsis is the systemic inflammatory response syndrome caused by infection, and kidney is one of the most often involving organs in sepsis.^1^, ^2^ Sepsis‐associated acute kidney injury (SA‐AKI) is a clinical critical illness, which is characterized by acute decline in renal function and has high morbidity and mortality in critically ill patients.^3^, ^4^ Studies have shown that inflammatory factors and oxidative stress damage lead to energy metabolism disorder and apoptosis of renal tubular epithelial cells (RTECs), which is one of the important factors result in SA‐AKI occurrence.^5^, ^6^ Therefore, exploring the potential molecular mechanism of RTECs injury is expected to provide new ideas for SA‐AKI treatment.

Bone marrow stem cells (BMSCs) are a kind of pluripotent stem cells belonging to the mesoderm, which have the potential of multi‐directional differentiation and play an important role in alleviating SA‐AKI.^7^ More and more studies have revealed that BMSCs mainly act through exosomes‐mediated paracrine pathway, which has shown great application potential in clinical treatment and drug delivery.^8^, ^9^ Previously, studies suggested that exosomes from artesunate‐treated BMSCs could facilitate osteogenesis by transferring SNHG7.^10^ Besides, luteolin‐loaded BMSCs‐derived exosomes might be a promising therapy for liver fibrosis.^11^ Therefore, loading BMSC‐derived exosomes with natural extracts may have favorable therapeutic effects on human diseases. Curcumin, the main active ingredient in the traditional Chinese medicine *Curcuma longa*, has been found to alleviate SA‐AKI.^12^, ^13^ However, whether curcumin‐loaded BMSCs‐derived exosomes mediates SA‐AKI process through inhibiting RTECs injury is not clear.

m6A is an important epigenetic modification that is thought to be involved in SA‐AKI progression.^14^ As a demethylase, fat mass and obesity‐associated protein (FTO) plays an important role in regulating body weight and fat content.^15^ Studies had revealed that silencing of FTO inhibited NLRP3 inflammasome through NF‐κB signaling in macrophages, thus reducing tissue damage and improving survival in LPS‐induced septic shock mice models.^16^ Moreover, FTO inhibited LPS‐induced HK2 cell autophagy and apoptosis via regulating miR‐372‐3p/ATG7 pathway by decreasing SNHG14 level through inhibiting its m6A modification.^17^ Importantly, it has been shown that BMSCs secrete FTO in exosome form.^18^ However, whether curcumin‐loaded BMSCs‐derived exosomes regulate FTO expression to mediate SA‐AKI has not been explored. Oxidative stress responsive 1 (OXSR1) is considered to be a regulator for SA‐AKI, which may contribute to LPS‐treated HK‐2 cell apoptosis, inflammation, and oxidative injury.^19^ By SRAMP website prediction, we found that OXSR1 had m6A methylation sites, and RBPsuit website predicted that there was a binding site between OXSR1 and FTO. But whether FTO regulates OXSR1 through m6A methylation modification to mediate SA‐AKI progression is unknown.

Here, we hypothesized that exosomes from curcumin‐induced BMSCs secreted FTO to reduce OXSR1 expression, which in turn inhibited RTECs injury. These studies may provide new way for SA‐AKI treatment.

## 2 MATERIALS AND METHODS

### 2.1 Cell culture, treatment and transfection

Human BMSCs (Procell, Wuhan, China) were cultured in specific BMSCs completed medium (CM‐H166, Procell). BMSCs were incubated with 10 μmol/L of curcumin (Sigma‐Aldrich, St. Louis, MO, USA) for 48 h to extract exosomes, with DMSO treatment as negative control.

Human RTECs (HK2; Procell) were cultured in MEM containing 10% FBS and 1% P/S. For mimic SA‐AKI cell model, HK2 cells were induced with 10 μg/mL LPS (Sigma‐Aldrich) for 24 h. For transfection, HK2 cells were transfected with siRNAs of FTO (si‐FTO), pcDNA FTO/OXSR1 overexpression vector, and negative controls (si‐NC and pcDNA) using Lipofectamine 3000 (Invitrogen, Carlsbad, CA, USA).

### 2.2 Exosome isolation and identification

After treated with curcumin or DMSO, the supernatants of BMSCs were collected for extracting exosomes (named as BMSCs‐EXO^Curcumin^ or BMSCs‐EXO^Control^) by differential ultracentrifugation. The morphological structure and particle size of BMSCs‐EXO were identified by transmission electron microscopy (TEM) and nanoparticle tracking analysis (NTA). The expression of exosome positive markers (CD9 and CD81) was measured by western blot (WB).

### 2.3 Exosome uptake detection

The isolated BMSCs‐EXO was incubated with Dio (10 μM, Beyotime, Shanghai, China) for 30 min. Then, HK2 cells were treated with 50 μg/mL of Dio‐labeled BMSCs‐EXO for 24 h. After counterstained with DAPI solution, the uptake of Dio‐labeled BMSCs‐EXO by HK2 cells was detected under a fluorescence microscope.

### 2.4 Western blot

Total proteins from exosomes, cells and mice kidney tissues were isolated by RIPA buffer (Beyotime) and moved to PVDF membranes after separated. Membrane was blocked and treated with antibodies (Abcam, Cambridge, CA, USA), including anti‐CD9 (1:1000, ab236630), anti‐CD81 (1:2000, ab109201), anti‐FTO (1:1000, ab124892), anti‐OXSR1 (1:3000, ab97694), anti‐GAPDH (1:2500, ab9485), and goat anti‐rabbit IgG (1:2000, ab205718). Finally, protein bands were visualized by ECL reagent (Beyotime). GAPDH was used as loading control.

### 2.5 Cell counting kit 8 assay

HK2 cells re‐seeded in 96‐well plates were cultured for 48 h. Then, cells were treated with cell counting kit 8 (CCK8) solution (Dojindo, Kumamoto, Japan), and cell viability was assessed using a microplate reader at 450 nm.

### 2.6 5‐Ethynyl‐2‐deoxyuridine assay

According to the instructions of 5‐ethynyl‐2‐deoxyuridine (EdU) assay kit (RiboBio, Guangzhou, China), HK2 cells seeded in 96‐well plates were incubated with EdU solution, TritonX‐100, Apollo solution, and DAPI solution. EdU positive cell rate was tested under a fluorescence microscope.

### 2.7 Flow cytometry

HK2 cells were collected and suspended with binding buffer. Then, cells were dyed with Annexin V‐FITC and PI solution (Beyotime). FACScalibur flow cytometer was used to analyze cell apoptosis rate.

### 2.8 Enzyme‐linked immunosorbent assay

Following the kit instructions, the levels of IL‐1β and TNF‐α in HK2 cell supernatant and mice serum samples were measured with IL‐β enzyme‐linked immunosorbent assay (ELISA) kit (Human PI305 or Mouse PI301, Beyotime) and TNF‐α ELISA kit (Human PT518 or Mouse PT512, Beyotime).

### 2.9 Detection of oxidative stress

According to kit instructions, the levels of SOD, MDA, and ROS in HK2 cells were detected by SOD assay kit (S0101S, Beyotime), MDA assay kit (ab118970, Abcam), and Cellular ROS assay kit (ab113851, Abcam), respectively.

### 2.10 Samples collection

On the premise of signed written informed consent, the peripheral bloods were obtained from 52 SA‐AKI patients and 52 healthy normal controls (underwent routine physical examination) at Affiliated Hospital of Jiangnan University. Serum samples were collected after centrifugation. Our study was approved by the Ethics Committee of the Affiliated Hospital of Jiangnan University.

### 2.11 Quantitative real‐time PCR

Total RNAs isolated from HK2 cells and serum samples were performed using TRIzol reagent (Invitrogen). After synthesized cDNA using cDNA Synthesis Kit (Takara, Tokyo, Japan), PCR was carried out using SYBR Green (Takara), cDNA and specific primers (Table 1). Fold changes of FTO and OXSR1 were analyzed by 2^−ΔΔCT^ method with GAPDH as normal control.

**TABLE 1:** Primer sequences used for qRT‐PCR.

Name	Primers for PCR (5′–3′)
OXSR1	
Forward	GGTCCATCAACAGGGACGAT
Reverse	TTAGGGGCACAATAAGCTGC
FTO	
Forward	TCTCATCTCGAAGGCAGGGA
Reverse	AAGGGGTATCGCCAAACCAG
GAPDH	
Forward	ATCACTGCCACCCAGAAGAC
Reverse	CCGTTCAGCTCAGGGATGAC

### 2.12 Methylated RNA immunoprecipitation assay

Basing on the instructions of methylated RNA immunoprecipitation (MeRIP) m6A kit (Millipore, Billerica, MA, USA), total RNAs isolated from HK2 cells transfected with or without si‐NC/si‐FTO/pcDNA/FTO were sheared into fragments, followed by incubated with Protein A/G magnetic beads pre‐coated with anti‐m6A/anti‐IgG. The immunoprecipitated RNA was collected to detect the m6A level of OXSR1 by qRT‐PCR.

### 2.13 RNA immunoprecipitation assay

After lysed with IP buffer (Millipore), HK2 cell lysates were treated by Protein A/G magnetic beads pre‐coated with anti‐FTO or anti‐IgG. Then, OXSR1 enrichment in immunoprecipitated RNA was determined by qRT‐PCR.

### 2.14 Cecal ligation puncture models

As previously described,^20^ SA‐AKI mice models were constructed by cecal ligation puncture (CLP) methods. Briefly, male C57BL/6 mice (VitalRiver, Beijing, China) were anesthetized with 2% pentobarbital sodium (50 mg/kg) by intraperitoneal injection. After exposed lower abdomen, the cecum of mice was ligated with a size 4 surgical suture. Then, the ligation site on both sides of the cecal wall was punctured with 18‐gauge needle twice to squeeze out a small amount of feces. Subsequently, the abdominal cavity of mice was sutured. Mice in the sham group underwent abdomen laparotomy and closure without CLP, and non‐treated mice were used as control group. Mice in the SA‐AKI + BMSCs‐EXO^Curcumin^ or SA‐AKI + BMSCs‐EXO^Control^ group were administered with BMSCs‐EXO^Curcumin^ or BMSCs‐EXO^Control^ (100 μg/mouse) via the tail vein 3 h after CLP surgery. After modeling 24 h, the blood samples of mice were taken from the orbital vein for obtaining mice serums. The levels of serum creatinine (Scr), blood urea nitrogen (BUN), and serum cystatin C (Scys C) were determined using an automated biochemistry analyzer (Beckman Coulter, Miami, FL, USA). Then, mice were euthanized, and the right kidney tissues were collected for histological examination and detecting FTO and OXSR1 protein expression by WB. Animal study was approved by the Animal Ethics Committee of the Affiliated Hospital of Jiangnan University (JN. No. 20240229N0200830[064]).

### 2.15 Histological examination

Paraffin‐embedded kidney tissues were cut into 4‐μm thick sections. Using hematoxylin and eosin staining kit (C0105M, Beyotime), sections were deparaffinized with xylene and stained with hematoxylin/eosin. For Masson staining, sections were stained with hematoxylin, Ponceau‐acid magenta, and bright green using Masson staining kit (C0189S, Beyotime). Subsequently, the histological morphology of mice kidneys were observed under a microscope.

### 2.16 Statistical analysis

All experiments were performed in triplicate, with each independent experiment set three times to generate an average value. Statistical analyses were carried out using GraphPad Prism 8.0 software. Data are expressed as the mean ± SD. Comparison between groups was conducted by Student's *t*‐test or ANOVA. *p* < 0.05 was considered statistical significance.

## 3 RESULTS

### 3.1 Exosomes from curcumin‐induced BMSCs alleviated LPS‐induced HK2 cell injury

**FIGURE 1:**
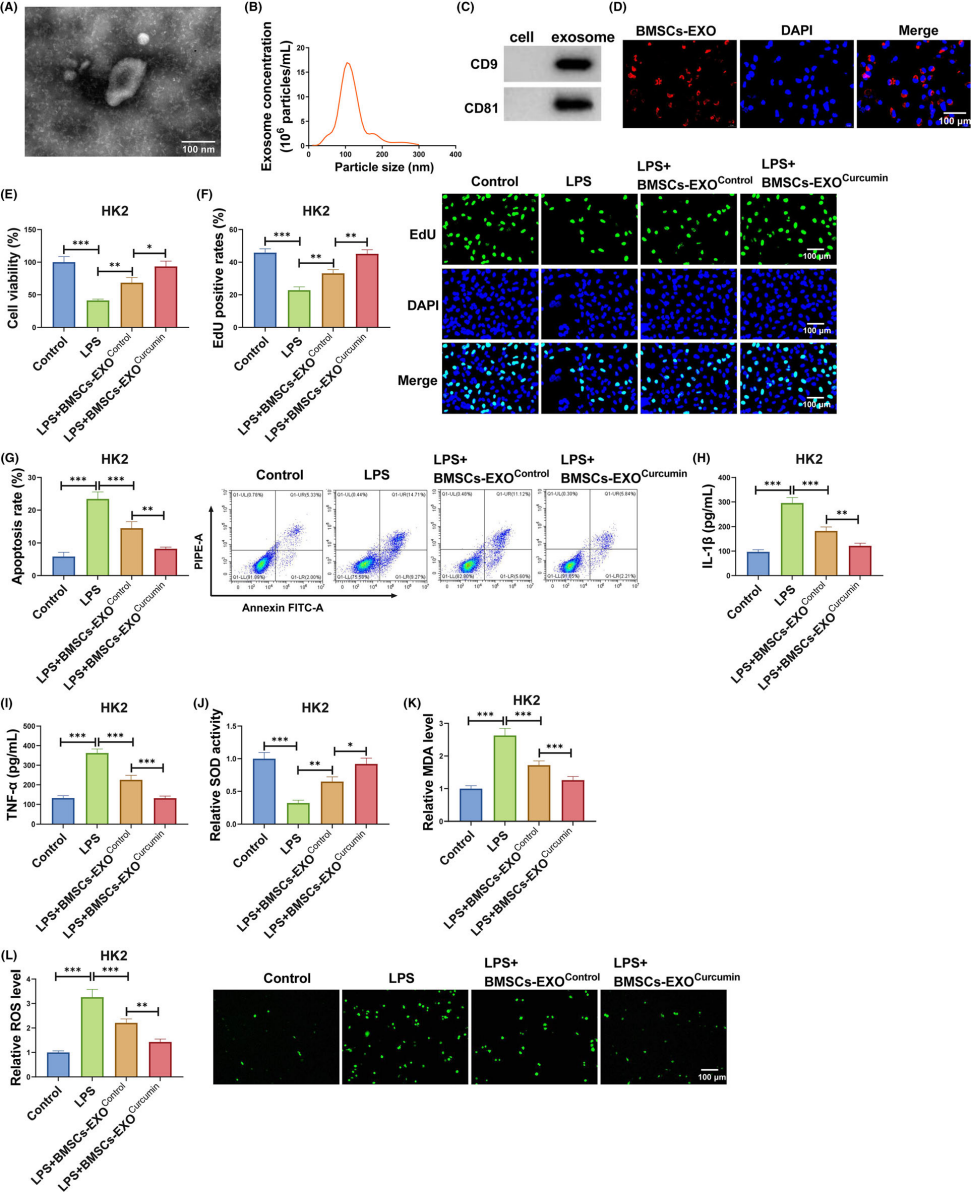
BMSCs‐EXO^Curcumin^ regulated LPS‐induced HK2 cell injury. The exosomes from BMSCs was identified by TEM (A) and NTA (B). (C) WB was used to detect CD9 and CD81 in BMSCs and BMSCs‐EXO. (D) Exosome uptake assay was used to confirm the uptake of BMSCs‐EXO in HK2 cells. (E–L) LPS‐induced HK2 cells were co‐cultured with BMSCs‐EXO^Control^ or BMSCs‐EXO^Curcumin^. CCK8 assay (E), EdU assay (F) and flow cytometry (G) were used to measure cell proliferation and apoptosis. (H,I) IL‐1β and TNF‐α levels were examined by ELISA. (J–L) SOD, MDA, and ROS levels were examined to assess oxidative stress. *p < 0.05, **p < 0.01, and ***p < 0.001.

Firstly, we isolated exosomes from the culture medium of BMSCs and observed the morphology and particle size of exosomes by TEM and NTA (Figure 1A,B). Also, the proteins of exosome markers (CD9 and CD81) were analyzed by WB (Figure 1C). These results confirmed the successful isolation of exosomes. To evaluate the delivery of BMSCs‐derived exosomes to HK2 cells, Dio‐labeled BMSCs‐EXO were incubated with HK2 cells. The results showed the presence of Dio labeling in recipient HK2 cells (Figure 1D), indicating that the labeled BMSCs‐EXO was delivered to HK2 cells. Then, LPS‐induced HK2 cells were co‐cultured with BMSCs‐EXO^Control^ or BMSCs‐EXO^curcumin^ to explore the effect of exosomes from curcumin‐induced BMSCs on LPS‐induced HK2 cell injury. BMSCs‐EXO^Control^ could promote viability and EdU positive rates in LPS‐induced HK2 cells, and the effect of BMSCs‐EXO^curcumin^ was more significant (Figure 1E,F). Also, BMSCs‐EXO^Control^ suppressed apoptosis rate, IL‐1β, TNF‐α, MDA, and SOD levels, while enhanced SOD activity in LPS‐induced HK2 cells. Furthermore, BMSCs‐EXO^curcumin^ was superior to BMSCs‐EXO^Control^ in inhibiting LPS‐induced HK2 cell apoptosis, inflammation, and oxidative stress (Figure 1G–L). Thus, we believed that exosomes from curcumin‐induced BMSCs might inhibit SA‐AKI process.

### 3.2 FTO expression in SA‐AKI patients, LPS‐induced HK2 cells, and BMSCs‐EXO^Curcumin^

**FIGURE 2:**
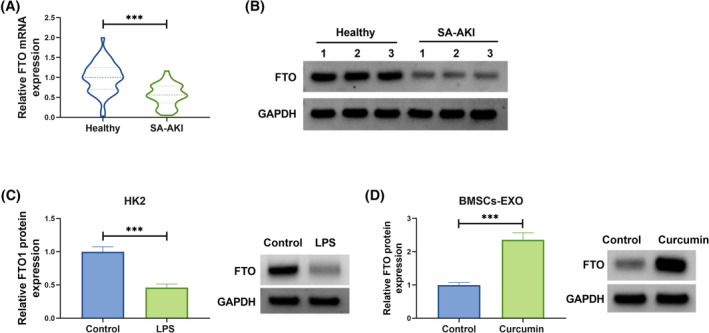
FTO expression in SA‐AKI patients, LPS‐induced HK2 cells and BMSCs‐EXO^Curcumin^. (A,B) FTO mRNA and protein levels were examined by qRT‐PCR and WB in the serum of SA‐AKI patients and healthy controls. (C) FTO protein level was detected by WB in HK2 cells treated with or without LPS. (D) FTO protein expression in BMSCs‐EXO^Control^ or BMSCs‐EXO^Curcumin^ was tested by WB. ***p < 0.001.

Because m6A methylation is a research hotspot, we were interested in m6A methylation in SA‐AKI and therefore speculated whether BMSCs‐derived exosomes regulate AKI through a methylation pathway. Among the selected five m6A methylation‐related genes, we found that FTO was significantly highly expressed in BMSCs‐EXO^Curcumin^ compared to BMSCs‐EXO^Control^ (Figure S1), so we chose FTO as the study object to explore whether curcumin‐derived exosomes from BMSCs regulate SA‐AKI by affecting the expression of FTO. In the serum of SA‐AKI patients, FTO mRNA and protein levels were lower than that in healthy controls (Figure 2A,B). Moreover, FTO protein expression was detected to be downregulated in LPS‐induced HK2 cells (Figure 2C). Through WB analysis, FTO protein expression was increased in BMSCs‐EXO^curcumin^ compared to BMSCs‐EXO^Control^ (Figure 2D). These data suggested that exosomal FTO from curcumin‐induced BMSCs might participate in SA‐AKI process.

### 3.3 Exosomal FTO from curcumin‐induced BMSCs relieved LPS‐induced HK2 cell injury

**FIGURE 3:**
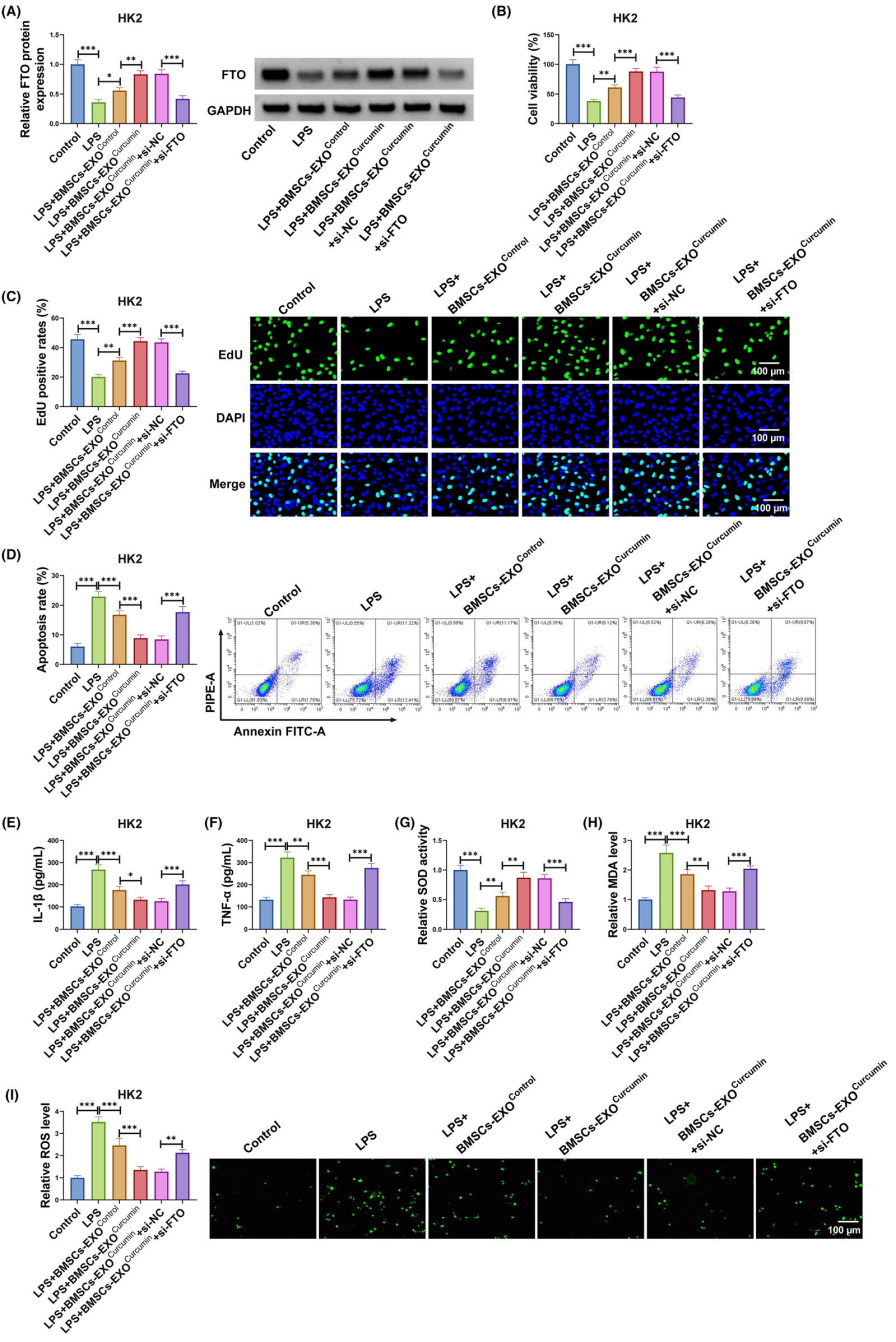
Exosomal FTO from BMSCs‐EXO^Curcumin^ regulated LPS‐induced HK2 cell injury. LPS‐induced HK2 cells were transfected with si‐NC/si‐FTO and co‐cultured with BMSCs‐EXO^Control^ or BMSCs‐EXO^Curcumin^. (A) FTO protein expression was measured by WB. Cell proliferation and apoptosis were examined using CCK8 assay (B), EdU assay (C) and flow cytometry (D). (E,F) ELISA was used to determine IL‐1β and TNF‐α levels. (G–I) Cell oxidative stress was assessed to detect SOD, MDA, and ROS levels. *p < 0.05, **p < 0.01, and ***p < 0.001.

To further explore whether exosomal FTO was involved in SA‐AKI process, LPS‐induced HK2 cells transfected with si‐FTO were co‐cultured with BMSCs‐EXO^curcumin^. The detection of FTO expression showed that BMSCs‐EXO^curcumin^ markedly increased FTO protein expression in LPS‐induced HK2 cells, and this effect was abolished by si‐FTO (Figure 3A). Besides, BMSCs‐EXO^curcumin^‐mediated the promoting on the viability and EdU positive rates in LPS‐induced HK2 cells could be reversed by FTO knockdown (Figure 3B,C). Moreover, the suppressive effect of BMSCs‐EXO^curcumin^ on apoptosis rate, IL‐1β, TNF‐α, MDA, and SOD levels, as well as the increasing effect on SOD activity, could be eliminated by FTO silencing in LPS‐induced HK2 cells (Figure 3D–I). These data confirmed that BMSCs‐EXO^curcumin^ ameliorated LPS‐induced HK2 cell injury by delivering FTO.

### 3.4 FTO inhibited OXSR1 expression through m6A demethylation modification

**FIGURE 4:**
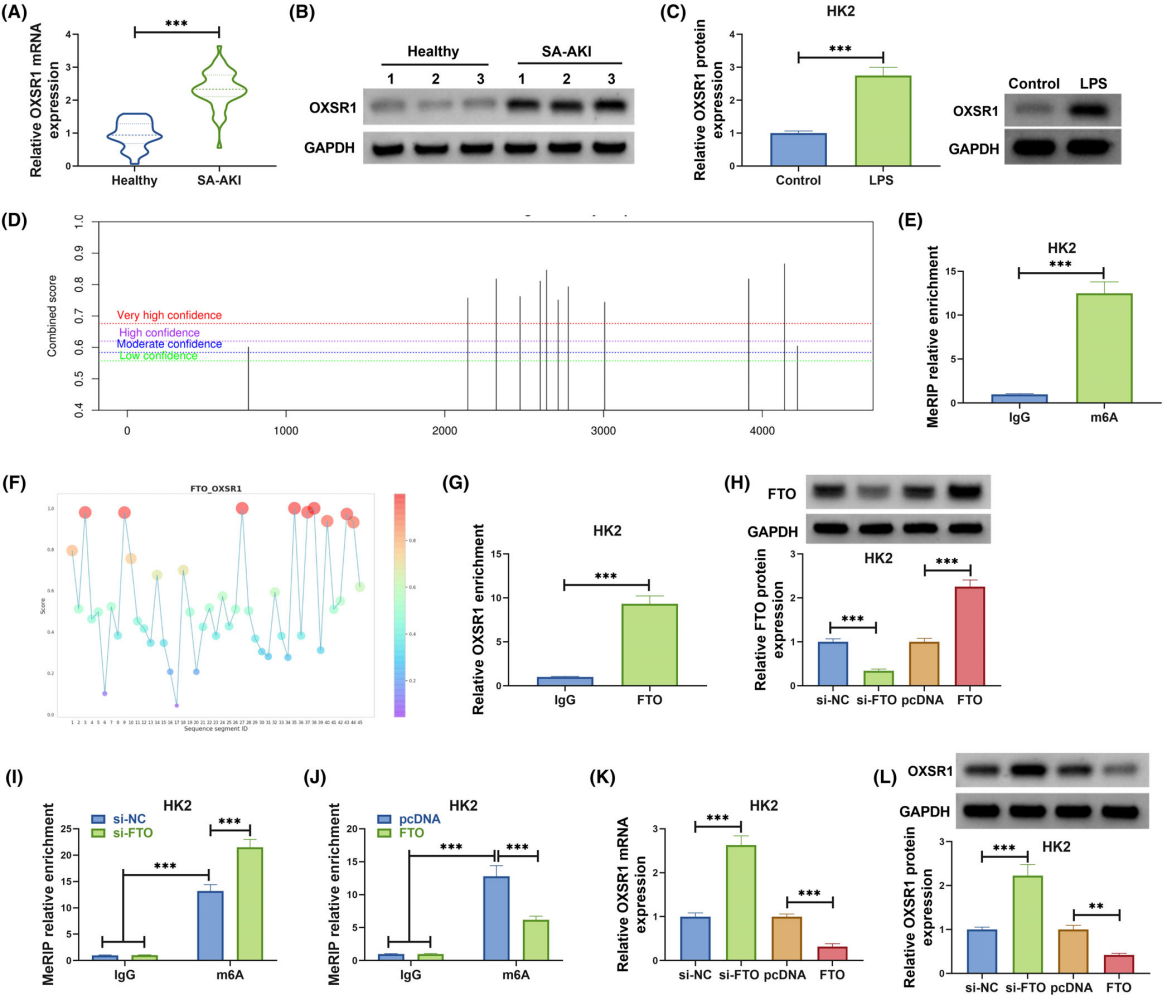
FTO regulated OXSR1 expression through m6A demethylation modification. (A,B) OXSR1 mRNA and protein levels in the serum of SA‐AKI patients and healthy controls were examined by qRT‐PCR and WB. (C) OXSR1 protein level in HK2 cells treated with or without LPS was detected by WB. (D) SRAMP website predicted the methylation modification sites of OXSR1. (E) MeRIP assay was used to detect the m6A level of OXSR1. (F) The binding sites between FTO and OXSR1 was predicted by RBPsuit website. (G) RIP assay was performed to confirm the interaction between FTO and OXSR1. (H) The transfection efficiencies of si‐FTO and FTO overexpression vector were confirmed by WB. (I,J) MeRIP assay was utilized to assess the effect of si‐FTO/FTO on the m6A level of OXSR1. (K,L) OXSR1 mRNA and protein levels were examined by qRT‐PCR and WB in HK2 cells transfected with si‐NC/si‐FTO/pcDNA/FTO. **p < 0.01 and ***p < 0.001.

OXSR1 mRNA and protein levels were upregulated in the serum of SA‐AKI patients compared with that in healthy controls (Figure 4A,B). Besides, we also detected high OXSR1 protein expression in LPS‐induced HK2 cells (Figure 4C). SRAMP website predicted that OXSR1 had methylation modification sites (Figure 4D), and MeRIP assay further confirmed that OXSR1 had enriched m6A level (Figure 4E). RBPsuit website predicted the binding sites of FTO and OXSR1 (Figure 4F). Further RIP assay results suggested that OXSR1 enrichment was increased by anti‐FTO (Figure 4G), confirming the interaction between them. To perform further analysis, we constructed si‐FTO and FTO overexpression vector to reduce and promote FTO protein expression in HK2 cells (Figure 4H). Through MeRIP assay, the m6A level of OXSR1 could be enhanced by FTO knockdown and suppressed by FTO overexpression (Figure 4I,J). In addition, we observed that FTO silencing significantly increased OXSR1 mRNA and protein levels, while FTO overexpression had an opposite effect (Figure 4K,L). Above results revealed that FTO demethylated OXSR1 to decrease its expression.

### 3.5 FTO inhibited LPS‐induced HK2 cell injury by downregulating OXSR1

**FIGURE 5:**
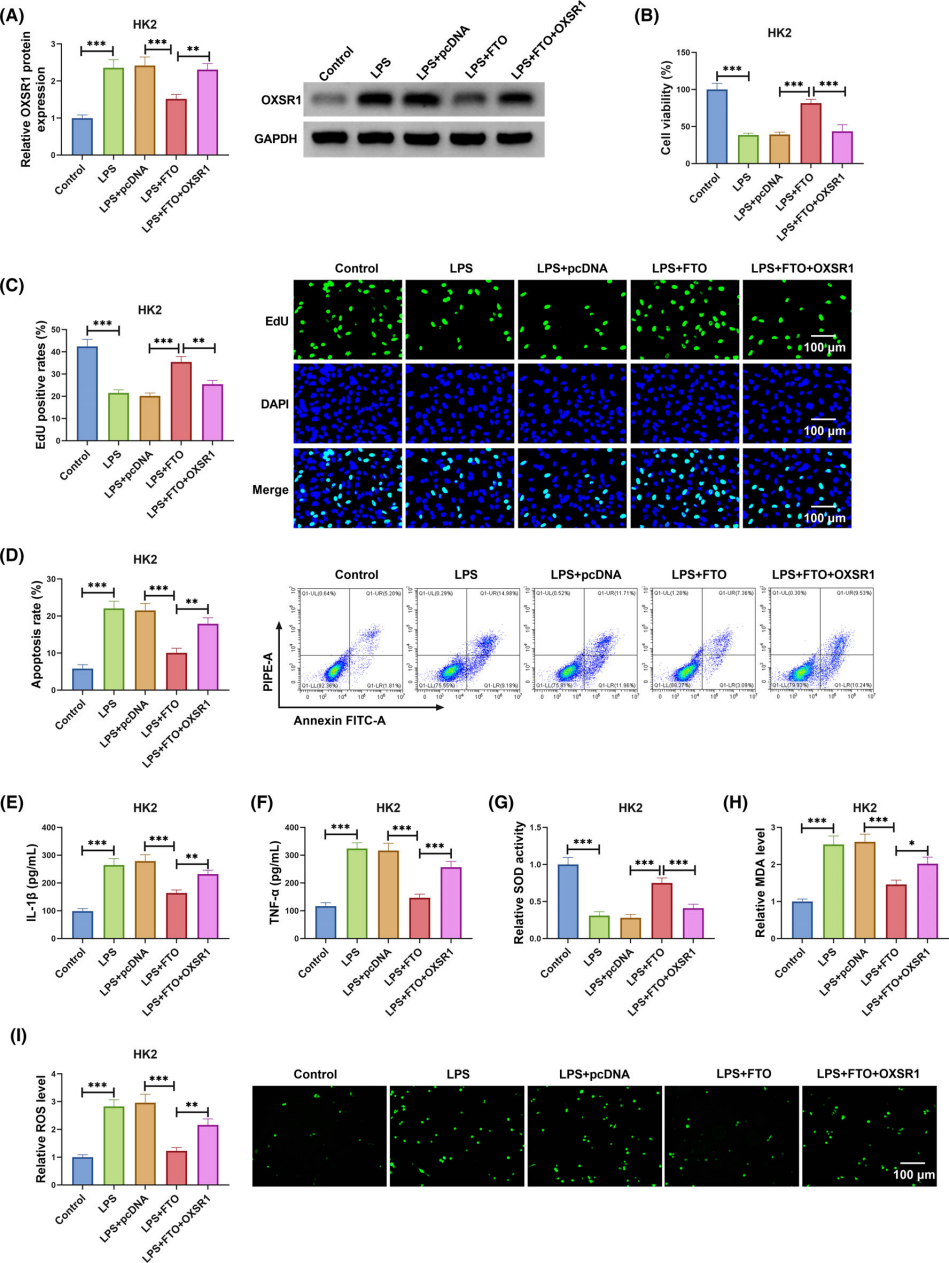
FTO/OXSR1 axis regulated LPS‐induced HK2 cell injury. LPS‐induced HK2 cells were transfected with pcDNA/FTO/OXSR1. (A) OXSR1 protein expression was measured by WB. CCK8 assay (B), EdU assay (C) and flow cytometry (D) were performed to determine cell proliferation and apoptosis. (E,F) IL‐1β and TNF‐α levels were measured by ELISA. (G–I) The levels of SOD, MDA, and ROS were detected to evaluate cell oxidative stress. **p < 0.01 and ***p < 0.001.

To confirm that FTO regulated SA‐AKI process by mediating OXSR1 expression, LPS‐induced HK2 cells were co‐transfected with FTO and OXSR1 overexpression vectors. Decreased OXSR1 expression caused by FTO could be eliminated by the transfection of OXSR1 overexpression vector in LPS‐induced HK2 cells (Figure 5A). As shown in Figure 5B,C, OXSR1 overexpression reversed the promotion effect of FTO on the viability and EdU positive rates of LPS‐induced HK2 cells. Besides, FTO overexpression suppressed apoptosis rate, IL‐1β, TNF‐α, MDA, SOD levels, and enhanced SOD activity in LPS‐induced HK2 cells, while these effects could be overturned by OXSR1 upregulation (Figure 5D–I). Therefore, FTO suppressed OXSR1 expression to alleviate SA‐AKI process.

### 3.6 Exosomes from curcumin‐induced BMSCs alleviated kidney injury in CLP‐induced SA‐AKI mice models

**FIGURE 6:**
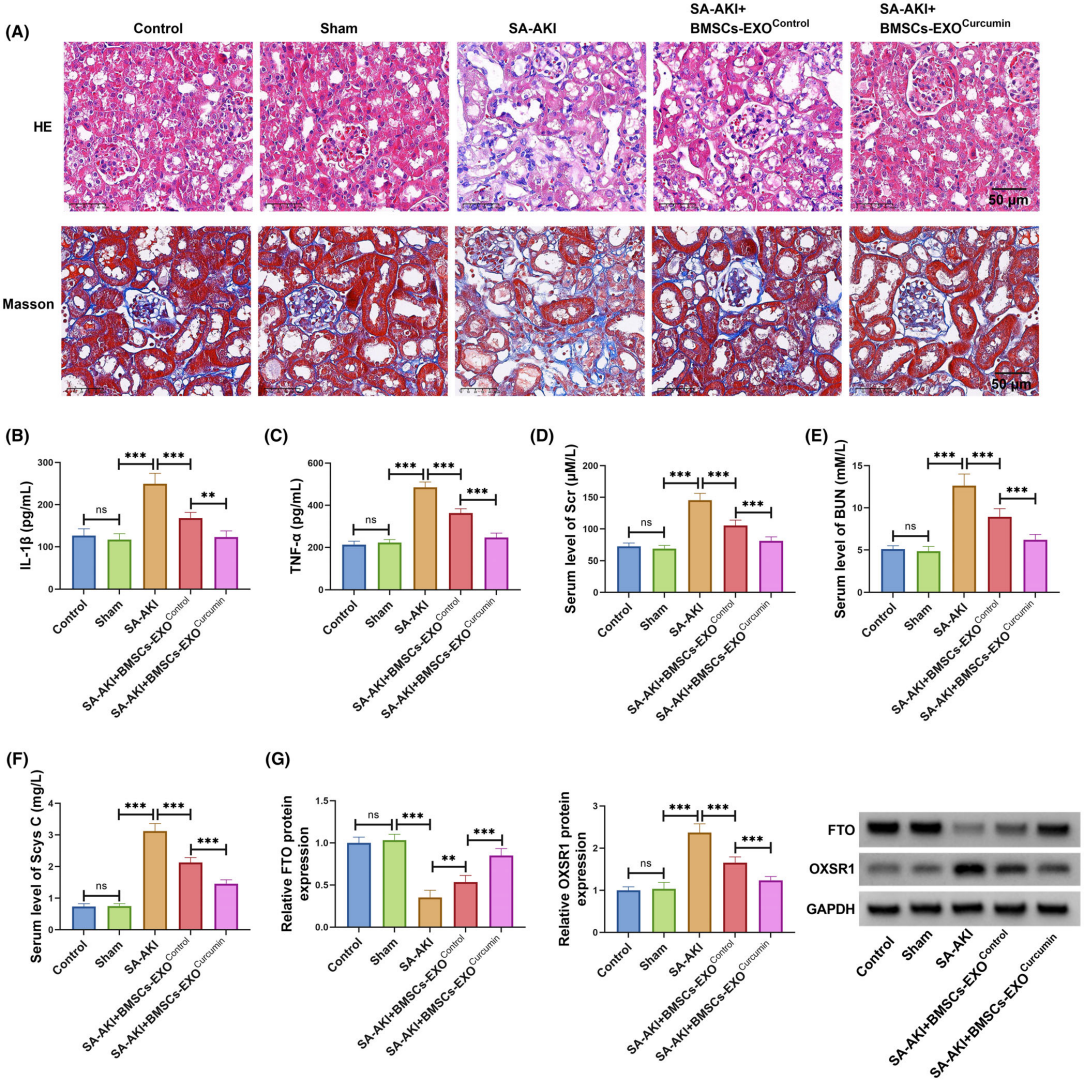
BMSCs‐EXO^Curcumin^ regulated kidney injury in CLP‐induced SA‐AKI mice models. CLP‐induced SA‐AKI mice models were injected with BMSCs‐EXO^Control^ or BMSCs‐EXO^Curcumin^. (A) H&E and Masson staining was used to assess kidney injury. (B,C) IL‐1β and TNF‐α levels in the serum of mice were measured by ELISA. (D–F) The serum levels of Scr, BUN, and Scys C in mice were used to assess kidney function. (G) FTO and OXSR1 protein levels in kidney tissues were examined by WB. **p < 0.01 and ***p < 0.001.

To further investigate the role of exosomes from curcumin‐induced BMSCs in SA‐AKI process, we constructed SA‐AKI mice models using CLP methods. Through H&E and Masson staining, we observed obvious glomerular swelling with a small amount of fibrosis and inflammatory cell infiltration in the SA‐AKI group, while these effects were improved in BMSCs‐EXO^Control^ group and more apparent in BMSCs‐EXO^Curcumin^ group (Figure 6A). Besides, the serum levels of IL‐1β, TNF‐α, Scr, BUN, and Scys C were significantly enhanced in the SA‐AKI group, while BMSCs‐EXO^Control^ could reduce their levels, and the effect of BMSCs‐EXO^Curcumin^ was more significant (Figure 6B–F). Additionally, BMSCs‐EXO^Control^ treatment increased FTO expression and decreased OXSR1 expression in CLP‐induced SA‐AKI mice models, and BMSCs‐EXO^Curcumin^ could further aggravate this effect (Figure 6G). Thus, exosomes from curcumin‐induced BMSCs improved kidney function in SA‐AKI mice models by regulating FTO/OXSR1 axis.

## 4 DISCUSSION

Exosomes are important mediators mediating intercellular communication, and exosomes‐contained RNA or protein can change the function of recipient cells.^21^, ^22^ BMSCs‐derived exosomes can not only mimic the biological functions of BMSCs, but also have more advantages than BMSCs in the application of disease treatment.^8^, ^23^ It had been reported that BMSCs‐derived exosomes could improve renal function and inhibit structural damage in LPS‐induced AKI mice models.^24^ Besides, BMSCs‐EXO ameliorated kidney injury in CLP‐induced SA‐AKI rats, as well as suppressed LPS‐induced HK2 cell inflammation and apoptosis.^25^ The evidence reveals the therapeutic potential of BMSCs‐EXO in SA‐AKI. Previous studies have shown that natural extracts‐loaded BMSCs‐EXO has great potential in disease treatment.^10^, ^11^ The anti‐inflammatory, anti‐oxidative, and anti‐tumor effects of curcumin, a natural extract, have been demonstrated in many diseases. Chen et al.^26^ suggested that curcumin administration could alleviate inflammatory injury of the lung and kidney in septic mice. Besides, curcumin reduced the secretion of inflammatory factors and the expression of oxidative stress‐associated protein to improve LPS‐induced liver injury.^27^ Not only that, curcumin has also been demonstrated to inhibit SA‐AKI progress.^12^, ^13^ Here, exosomes (BMSCs‐EXO^Curcumin^) were isolated from BMSCs cells treated with curcumin to observe its effects on LPS‐induced HK2 cell injury. BMSCs‐EXO could suppress LPS‐induced cell apoptosis, inflammation, and oxidative stress, and the effect of BMSCs‐EXO^Curcumin^ was better than that of BMSCs‐EXO^Control^. These evidences suggest the utility of exosomes from curcumin‐induced BMSCs in alleviating the progression of SA‐AKI.

As m6A eraser, FTO mainly plays demethylation activity for the m6A of single‐stranded RNA, thus regulating gene expression.^28^, ^29^ Huang et al.^30^ revealed that FTO suppressed nasopharyngeal carcinoma cell ferroptosis to enhance radioresistance via stabilizing the interaction between OTUB1 and SLC7A11 by inhibiting the m6A modification of OTUB1. Besides, Yu et al. showed that FTO decreased the m6A modification of cGAS to reduce its mRNA stability, thereby inactivating Sting/NF‐κB signaling and alleviating cerebral ischemia/reperfusion‐induced neuroinflammation.^31^ FTO was underexpressed in LPS‐induced cardiomyoblasts, and its decreased expression might be related to endotoxemia‐induced myocardial inflammation and dysfunction.^32^ FTO is considered to be a biomarker for the clinic diagnose of sepsis.^33^ In sepsis‐induced cardiac injuries, FTO inhibited ferroptosis to relieve heart inflammation and dysfunction in mice by regulating BACH1 expression.^34^ Although it has been confirmed that FTO has anti‐SA‐AKI function,^17^ but its more role remains to be revealed. Consistent with previous study,^17^ we detected the low FTO expression in patients and cell models. Also, we determined that FTO was highly expressed in BMSCs‐EXO^Curcumin^ more than in BMSCs‐EXO^Control^, which was consistent with the previous conclusion of Xu et al.^18^ that FTO was existed in exosomes‐derived from BMSCs. Further analysis showed that FTO knockdown reversed the suppressive effect of BMSCs‐EXO^Curcumin^ on LPS‐induced HK2 cell injury, confirming that exosomes from curcumin‐induced BMSCs might inhibit SA‐AKI process by secreting FTO.

OXSR1 had pro‐oxidant and pro‐apoptosis in neurons to aggravate epilepsy progression.^35^ It had been reported that decreased OXSR1 expression could improve myocardial damage and inflammation in septic mice models.^36^ In sepsis‐induced acute lung injury, OXSR1 overexpression promoted LPS‐induced inflammation and apoptosis in airway epithelial cells.^37^ Besides, OXSR1 might be a potential treatment target for SA‐AKI, which overexpression could promote LPS‐induced HK2 cell inflammation.^38^ Also, downregulation of OXSR1 might be related to the reduced apoptosis, inflammation, and oxidation in LPS‐treated HK2 cells and CLP‐induced rat model.^39^ Moreover, OXSR1 overexpression decreased apoptosis and the secretion of TNF‐α and IL‐6 in LPS‐treated HK‐2 cells.^40^ The above studies confirm the positive role of OXSR1 in the progression of SA‐AKI. Consistent with above studies, we detected the high OXSR1 expression in patients and cell models. By analysis, we identified that OXSR1 had methylation modification sites and could interact with FTO. Further analysis revealed that FTO could inhibit OXSR1 expression by reducing its m6A level. The reversal effect of OXSR1 on FTO‐mediated anti‐apoptosis, anti‐oxidant, and anti‐inflammation in LPS‐induced HK2 cells confirmed that FTO might restrain SA‐AKI process by reducing OXSR1 expression. Importantly, we determined that BMSCs‐EXO^Curcumin^ decreased OXSR1 expression in SA‐AKI mice models, further verifying the existence of BMSCs‐EXO^Curcumin^/FTO/OXSR1 axis. Previously, studies showed that OPG/RANKL/RANK/TLR4 pathway was involved in the pathogenesis of SA‐AKI.^20^ Here, our study proposes a novel mechanism for the regulation of SA‐AKI, namely the BMSCs‐EXO^Curcumin^/FTO/OXSR1 pathway, which provides new insights into the development of SA‐AKI.

**FIGURE 7:**
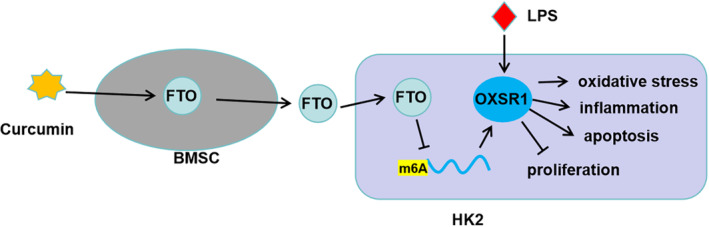
Mechanistic diagram of the present study. Exosomal FTO from curcumin‐induced BMSCs to alleviate LPS‐induced HK2 cell oxidative stress, inflammation and apoptosis by reducing OXSR1 expression.

In conclusion, our data showed that curcumin‐induced exosomal FTO from BMSCs alleviated LPS‐induced HK2 cell injury and kidney injury in CLP mice models by reducing the m6A methylation of OXSR1 (Figure 7). This research is the first to reveal the favorable role of exosomes from curcumin‐induced BMSCs in combating SA‐AKI. These findings provided new evidence for the use of BMSCs‐derived exosomes in SA‐AKI treatment, and the proposal of FTO/OXSR1 axis offers a potential molecular target for SA‐AKI.

## CONFLICT OF INTEREST STATEMENT

The authors declare no conflicts of interest.

## Supporting information


**FIGURE S1.** BMSCs‐EXO^Curcumin^ regulated FTO mRNA expression. qRT‐PCR was used to detect METTL3, METTL14, WTAP, FTO, and ALKBH5 mRNA expression in BMSCs‐EXO^Curcumin^ and BMSCs‐EXO^Control^. ***p < 0.001.

## Data Availability

The data that support the findings of this study are available from the corresponding author upon reasonable request.
