# Supplementary figures and images for: Curcumin‐induced exosomal FTO from bone marrow stem cells alleviates sepsis‐associated acute kidney injury by modulating the m6A methylation of OXSR1
Source: Kaohsiung J Med Sci. 2024 Dec 30;41(2):e12923. doi: 10.1002/kjm2.12923 (PMC11827542; doi:10.1002/kjm2.12923)

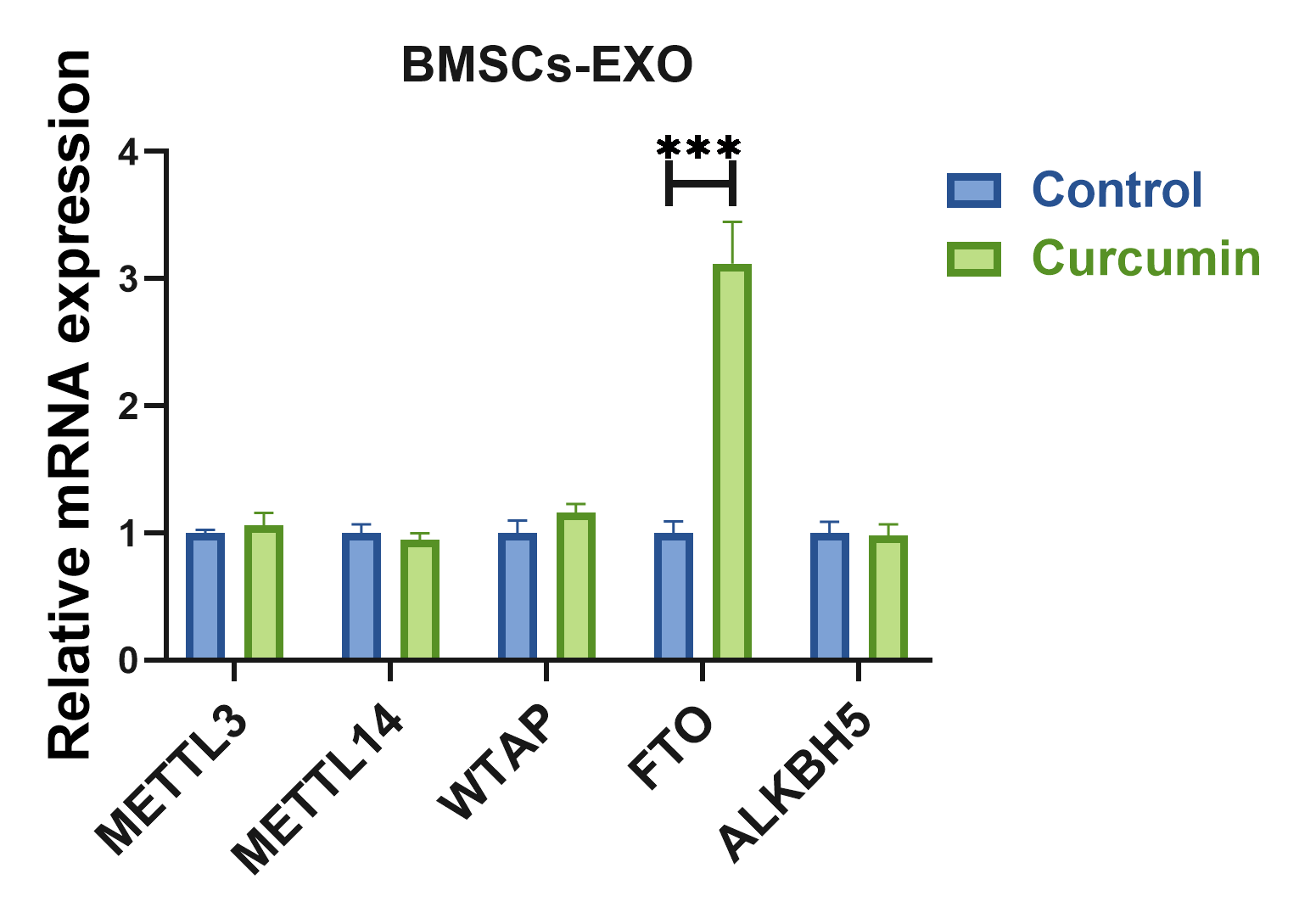

Supplement: Supplementary file 1 — FIGURE S1. BMSCs‐EXOCurcumin regulated FTO mRNA expression. qRT‐PCR was used to detect METTL3, METTL14, WTAP, FTO, and ALKBH5 mRNA expression in BMSCs‐EXOCurcumin and BMSCs‐EXOControl. ***p < 0.001. [file KJM2-41-e12923-s001.tif]
